# Disseminated intracranial xanthoma disseminatum: a rare case report and review of literature

**DOI:** 10.1186/s13000-016-0531-1

**Published:** 2016-08-17

**Authors:** Guang-Zhi Yang, Jing Li, Lu-Ping Wang

**Affiliations:** Department of Pathology, the General Hospital of Beijing Military Command of PLA, Nan Men Cang, No. 5, Dong Si, Dongcheng District, Beijing, 100700 People’s Republic of China

**Keywords:** Xanthoma disseminatum, Disseminated, Histiocytic neoplasm, MR, Immunohistochemistry

## Abstract

**Background:**

Xanthoma disseminatum (XD) is a rare benign histiocytic proliferating disease of non-Langerhans cell origin, which is clinically mainly characterized by cutaneous or mucous lesions. Although XD is acknowledged of one systematic disease, involvement of the central nervous system is quite rare.

**Case presentation:**

We presented one 34-year-old Chinese female with disseminated intracranial XD without cutaneous or oral mucosal papules and masses of the other organs. MR imaging displayed multiple heterogeneous masses with intense enhancement in the right frontal lobe, temporal lobe, corpus callosum, left cuneus, suprasellar region, and right cerebellum. Pathological examination showed a neoplastic lesion composed of plentiful epitheloid or spindle cells. The cell had pink cytoplasm of vacuolation and foam with deviated nucleus absent of atypia and mitosis. The histiocytic markers including CD163, CD11c, Mac387 and CD68 were positive, whereas S-100, CD1a, GFAP, CD21, CD23 and so on were negative immunohistochemically.

**Conclusions:**

Intracranial XD without systemic involvement was extremely rare, which was supposed to be considered in differential diagnosis with other neoplasms of histiocytic origin or gliomas.

## Background

Xanthoma disseminatum (XD) is a rare benign histiocytic proliferating disease of non-Langerhans cell origin [1, 2]. It is clinically mainly characterized by a number of symmetrically distributed cutaneous yellow-brown papules, often affecting faces, trunk and limbs. XD may also involve the mucous membranes of conjunctivae, lips, tongue, cheeks, gingiva, palate, and so on. Respiratory tract, including the pharynx, larynx, trachea and bronchi, is sometimes involved. In addition, involvement of the viscera has been occasionally reported [3–5]. Although XD is always a systemic disease [3], involvement of the central nervous system (CNS) is quite rare, and until now less than 100 cases in the literature written in English have been reported. In our medical practice, we experienced with one rare case of intracranial XD without involvement of other organs. Herein, we presented the case and discussed with review of the literature.

## Case presentation

**Fig. 1:**
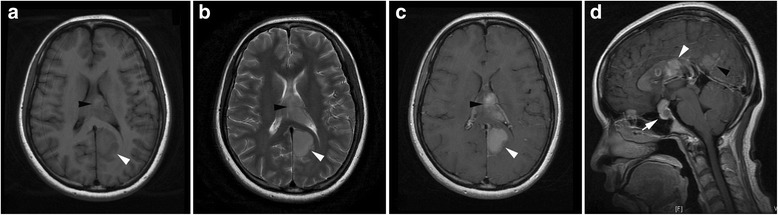
Axial T1 and T2-weighted images showed mass lesions in callosum (*black arrowhead*), left occipital lobe (*white arrowhead*), saddle area and anterior skull base (*white arrow*) respectively, which displayed enhancement in enhanced axial T1 and sigttal T1-weighted images. **a** Axial T1WI. **b** Axial T2WI. **c** Enhanced axial T1WI. **d** Enhanced sigttal T1WI

**Fig. 2:**
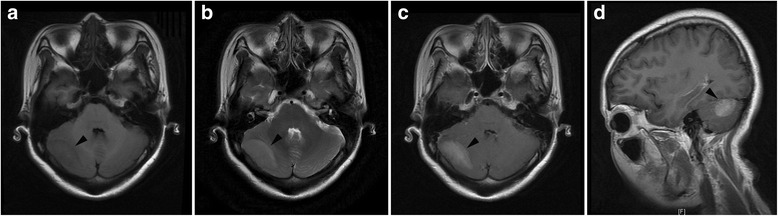
Axial T1 and T2-weighted images showed mass lesions in right cerebellum (*black arrowhead*), which displayed enhancement in enhanced axial T1 and sigttal T1-weighted images. **a** Axial T1WI. **b** Axial T2WI. **c** Enhanced axial T1WI. **d** Enhanced sigttal T1WI

One 34-year-old Chinese female presented with 1-month history of dizziness, nausea, and vomiting, and followed by difficulty in swallowing and walking instability. Physical examinations revealed no significant abnormality. Laboratory tests showed that results of complete blood count and biochemical profiles were roughly normal. Brain magnetic resonance (MR) imaging displayed multiple heterogeneous masses with intense enhancement in the right frontal lobe, temporal lobe, corpus callosum, left cuneus, suprasellar region, and right cerebellum (Figs. 1 and 2). Diagnosis of lymphoma was preferred and biopsy was performed in the right cerebellum then.

The samples obtained at surgery were fixed in 10 % buffered formalin and embedded in paraffin for pathological study. Sections were stained by Hematoxylin-Eosin for routine histopathological investigation. Immunohistochemical analysis was performed by steam heat-induced epitope retrieval and the Dako Envision Detection System.

**Fig. 3:**
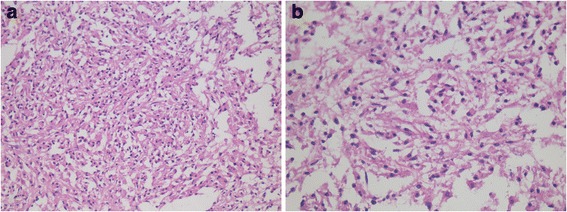
The tumor was mainly composed of epitheloid or spindle cells with numerous pink cytoplasm of vacuolation and foam. **a** HE 200× magnification. **b** HE 400× magnification

Pathological examination showed a neoplastic lesion composed of plentiful epitheloid or spindle cells instead of the normal cerebellum (Fig. 3). The cells had numerous pink cytoplasm of vacuolation and foam with deviated nucleus. Atypia and mitotic figures were nearly absent. There were also a few mature lymphocytes infiltrating, especially around focal vessels.

**Fig. 4:**
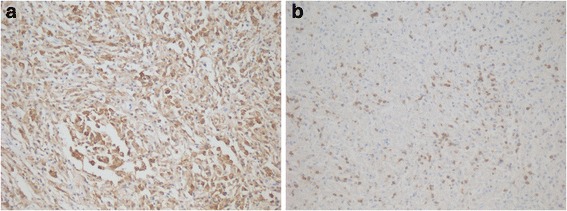
Immunohistochemical studies revealed that the tumor cells were positive for CD163 (**a**), and CD3 was scattered positive for the small T lymphocytes (**b**)

Immunohistochemical studies displayed that CD163, CD11c, Mac387 and CD68 (both KP1 and PGM clones) were diffusely positive, which verified that the neoplasm cells were of histiocytic origin (Fig. 4a). CD3 was scattered positive for the small T lymphocytes (Fig. 4b). Other markers, including S-100, CD1a, glial fibrillary acidic protein (GFAP), oligo-2, CD21, CD23, CD35, CD30, CD20, Melan-A and HMB45, were all negative, which excluded Langerhans cell histocytisis (LCH), glioma, lymphoma, and follicular dendritic cell sarcoma, and so on. The pathological diagnosis was disseminated intracranial XD.

In the comprehensive examinations during the preoperative preparation, no mass in the internal organs, including the liver, gallbladder, kidneys, pancreas, bladder, uterus, and ovary examined by B ultrasound and lungs scanned by computed tomography (CT), was found. No cutaneous or oral mucosal papules were found after careful consultation from the dermatologist since XD was diagnosed morphologically. So diagnosis of intra-axial brain XD without systemic involvement was established.

The patient hadn’t received any treatments after surgery. Follow-up for 1 year showed that the lesions had still kept stable.

## Discussions

XD, also named as Montgomery syndrome, was first described by Von Graefe in 1867, and then reviewed and credited by Virchow in 1871 [6]. It is reported that XD affected populations aged from 5 months to 70 years old with most before 25 years old. It is more prevalent in males with a male-to-female ratio of 2:1 [7]. No typical inheritance pattern is confirmed. XD is a histiocytic disorder of non-Langerhans cell origin morphologically characterized by xanthomatous deposits in the absence of lipid metabolism disorder and hyperlipidemia [8].

Although it is widely acknowledged that XD is a systematic disease, involvement of CNS is quite rare [9]. Clinical manifestations of patients with CNS XD varied greatly according to the involving positions, including headache, nausea, vomiting, dizziness, seizures, ataxia, or visual field defects. Hammond and Mackenzie reviewed the literatures and summarized that less than 100 cases of CNS XD had been reported [6]. XD always affects the dura mater, which is actually extra-axial [10]. The first axial case located in the pontine was reported by Chiari [6]. Most cases involving the parenchymal CNS are located in the pituitary or hypothalamus, whereas the number of the outside is less than 5 % [11]. It is believed that besides the pituitary and hypothalamus, the brainstem and cerebellum were always involved because of the local environment such as the blood–brain barrier and short distance from the ventricle or meninges [6].

The exact diagnosis of XD is relied on pathological examination, and other neoplasms of histiocytic origin such as LCH and Rosai-Dorfman disease (RDD) are under differential diagnosis. Typical histopathological manifestations of XD are large pleomorphic cells with cytoplasmic bubbles, which can merge and form multinucleated Toutan giant cells. Inflammation is not striking, and varying number of mature T lymphocytes is mixed in the lesion. By immunohistochemistry, the tumor cells are positive for histiocytic markers including CD68, CD163, CD11c and so on, whereas negative for S-100 protein and CDla. Eosinophils are one of components in LCH, and nuclear grooves are the prominent feature of the tumor cells. In addition, immunohistochemical S-100 and CDla are positive, and Birbeck bodies are detected under electron microscopic examination, which can be identified from XD. Inflammatory background is striking in RDD, and the tumor cells are remarkably huge with intracytoplasmic small lymphocytes swallowed and immunohistochemically S-100 positive. Sometimes, intraparenchymal XD is also supposed to be distinguished from Pleomorphic xanthoma astrocytoma (PXA), and immunohistochemical staining of GFAP is crucial [12].

Since XD is always one systemic disease, cutaneous or mucous biopsy is supposed to perform before diagnosis. Serum lipid examination is also necessary. However, our case is quite distinct, which multiple locations of CNS were involved and no other differences were founded, including skin or mucous papules, masses of the other organs besides serum lipid.

According to natural course and outcome, XD was divided into three clinical types by Caputo [13]. The most common is persistent form, followed by progressive form with systemic involvement, and the rare is regression form. Thus, XD seems to have self-healing tendency. Besides, as far as XD with intracranial involvement is concerned, prognosis is also closely related with the involving location. Hammond and Mackenzie reported that fatality rate of XD patients with the pituitary/the hypothalamus was 63 %, and yet that of the posterior fossa was 100 % [11]. For cases with intracranial XD, symptoms progress is supposed to be observed and mass change should be reviewed by MR imaging to determine the clinical types and following therapies. The present case was identified of persistent form in the follow-up, thus operation seemed unnecessary, and yet no accurate diagnosis was established until operation.

Treatments for intracranial XD remain determined because of limited cases and paradoxical results [11, 14]. Steroids or combined with chemotherapy drugs were usually adopted for cases with intracranial wide-spread or large mass. It is generally believed that the radiotherapy had no satisfactory effect for intracranial XD, but multi-mode radiation may have some effect. Surgery was always the choice for localized XD. So, it is easy to see different strategies for different cases, but dynamic observation is the premise. In principle, clinical treatments such as surgery or chemotherapy were performed for the cases with progress or large volume, dynamic observation was taken for the cases ofself-limited tendency or slow progress without obvious symptoms.

## Conclusions

Here, we presented the case of intracranial XD without systemic involvement. MR imaging displayed multiple heterogeneous masses with intense enhancement. Pathological examination showed a neoplasm composed of plentiful epitheloid or spindle cells with vacuolated and foamy pink cytoplasm. The tumor cells were absent of atypia and positive for histiocytic markers immunohistochemically. Such case was extremely rare, which was supposed to be considered in differentiation with other neoplasms of histiocytic origin or gliomas.

## Abbreviations

CNS, central nervous system; CT, computed tomography; GFAP, glial fibrillary acidic protein; LCH, langerhans cell histocytisis; MR, magnetic resonance; PXA, pleomorphic xanthoma astrocytoma; RDD, rosai-dorfman disease; XD, xanthoma disseminatum
